# Pharmacomicrobiomics of Antidepressants in Depression: A Systematic Review

**DOI:** 10.3390/jpm13071086

**Published:** 2023-06-30

**Authors:** Lisa C. Brown, William V. Bobo, Cory A. Gall, Daniel J. Müller, Chad A. Bousman

**Affiliations:** 1 Great Scott! Consulting LLC, New York, NY 11222, USA; 2 Department of Psychiatry & Psychology, Mayo Clinic, Jacksonville, FL 32224, USA; 3 Department of Veterinary Tropical Diseases, University of Pretoria, Onderstepoort 0028, South Africa; 4 Pharmacogenetics Research Clinic, Campbell Family Mental Health Research Institute, Centre for Addiction and Mental Health, Toronto, ON M6J 1H4, Canada; 5 Department of Psychiatry, University of Toronto, Toronto, ON M6J 1H4, Canada; 6 Department of Psychiatry, Psychosomatics and Psychotherapy, Center of Mental Health, University Hospital of Würzburg, 97080 Würzburg, Germany; 7 The Mathison Centre for Mental Health Research and Education, Hotchkiss Brain Institute, Cumming School of Medicine, University of Calgary, Calgary, AB T2N 4N1, Canada; chad.bousman@ucalgary.ca; 8 Departments of Medical Genetics, Psychiatry, Physiology and Pharmacology, and Community Health Sciences, University of Calgary, Calgary, AB T2N 4N1, Canada; 9 Alberta Children’s Hospital Research Institute, University of Calgary, Calgary, AB T2N 4N1, Canada

**Keywords:** pharmacomicrobiomics, antidepressants, microbiota, psychiatry, precision medicine

## Abstract

This systematic review evaluated the animal and human evidence for pharmacomicrobiomics (PMx) interactions of antidepressant medications. Studies of gut microbiota effects on functional and behavioral effects of antidepressants in human and animal models were identified from PubMed up to December 2022. Risk of bias was assessed, and results are presented as a systematic review following PRISMA guidelines. A total of 28 (21 animal, 7 human) studies were included in the review. The reviewed papers converged on three themes: (1) Antidepressants can alter the composition and metabolites of gut microbiota, (2) gut microbiota can alter the bioavailability of certain antidepressants, and (3) gut microbiota may modulate the clinical or modeled mood modifying effects of antidepressants. The majority (n = 22) of studies had at least moderate levels of bias present. While strong evidence is still lacking to understand the clinical role of antidepressant PMx in human health, there is evidence for interactions among antidepressants, microbiota changes, microbiota metabolite changes, and behavior. Well-controlled studies of the mediating and moderating effects of baseline and treatment-emergent changes in microbiota on therapeutic and adverse responses to antidepressants are needed to better establish a potential role of PMx in personalizing antidepressant treatment selection and response prediction.

## 1. Introduction

The clinical response to antidepressants in individuals with major depressive disorder (MDD) is highly variable, both with respect to effectiveness and adverse effects [1]. Notably, only 50% of individuals respond to their first antidepressant while nearly one-third of patients with MDD fail to achieve meaningful benefit after several therapeutic antidepressant trials [2,3]. Variables linked to antidepressant response include family history, genetic profile, socioeconomic position, educational attainment, age of depression onset, psychiatric and general medical comorbidity, substance use disorders, co-occurring anxiety, duration of depressive episodes, and lifestyle factors [4]. However, none of these factors, individually, are sufficiently valid for clinical use as response predictors [5]. Integrating omics-based biomarkers with clinical features is a promising approach to improving clinical response prediction in patients with MDD [6,7]. Although integrating pharmacogenomics with measurement-based clinical decision making can increase the chances of achieving symptomatic remission in individuals with MDD who are treated with antidepressants [8], additional biomarkers for therapeutic response and adverse effects are needed. 

An emerging area of research into response to antidepressants lies in the gut, aptly named pharmacomicrobiomics (PMx), which in this case describes how the microbiome may potentiate or limit the effects of antidepressants in MDD [9]. Animal and human studies have evaluated how changes in the gut microbiome and gut microbial metabolites may influence risk for various diseases, including psychiatric diseases, as well as clinical outcomes of treatment for these conditions [10,11]. While the application of PMx in mood disorders is in its relative infancy, an increasing number of studies have reported differences in gut microbial diversity and richness between individuals with MDD compared to healthy controls [11,12] and a relative overabundance of inflammatory microbes in depressed individuals, supporting a gut-inflammatory-brain hypothesis for depression [13,14]. Moreover, prior research has linked specific gut microbiota with depression severity and gut microbiota richness and diversity with remission from depression [15]. Preclinical studies have shown that depressive behaviors can be induced by transplanting fecal microbiota from depressed individuals into germ-free rats [16] and by disrupting gut microbiota with exposure to antibiotics [17], that latter of which may be accompanied by increased inflammatory responses, further supporting a role of microbiota-induced inflammation in mood disorders [18]. 

There is emerging evidence to suggest that antidepressants interact with the gut microbiome to exert therapeutic effects. In preclinical studies, several conventional monoamine-based antidepressants have demonstrated antimicrobial or gut microbiota-altering effects [19,20]. Preclinical studies have also suggested that antidepressive-like effects of racemic ketamine and ketamine metabolites may be facilitated by the microbiota-gut-brain axis and that ketamine may also help regulate the composition of microbiota in the gut [21]. As highlighted in this review, much less is understood about how variations in the microbiota-gut-brain axis affect the pharmacokinetics and clinical response to antidepressants in depressed patients, including patients with immune-related depression [9]. As the sequencing of genetic material from gut microbes becomes easier and less-expensive, PMx may emerge as an increasingly important tool for precision prescribing of antidepressants in patients with MDD. Herein, we review the current knowledge in the field of PMx of antidepressants for depression in both animal models and human studies.

## 2. Materials and Methods

### 2.1. Information Sources and Search Strategy

A systematic search of PubMed (to present, including Epub Ahead of Print, In-Process Non-Indexed Citations, and Daily) was performed on 18 December 2022. The following search strategy was deployed: ((antidepressant OR selective serotonin reuptake inhibitor OR SSRI OR serotonin norepinephrine reuptake inhibitors OR monoamine oxidase inhibitor OR TCA OR Tricyclic antidepressant OR SNRI OR MAOI OR mirtazapine OR bupropion OR vilazodone OR vortioxetine OR nefazodone OR sertraline OR paroxetine OR citalopram OR escitalopram OR fluoxetine OR clomipramine OR imipramine OR trimipramine OR desipramine OR amitriptyline OR nortriptyline OR doxepin OR protriptyline OR amoxapine OR duloxetine OR desvenlafaxine OR venlafaxine OR levomilnacipran OR milnacipran OR trazodone) AND (depression or major depressive disorder OR healthy OR MDD) AND (microbiome OR microbiota OR pharmacomicrobiomics OR psychopharmacomicrobiomics or psychobiotic)). The systematic review followed the 2020 Preferred Reporting Items for Systematic Reviews and Meta-Analyses (PRISMA) reporting recommendations [22]. 

### 2.2. Eligibility Criteria

Original animal and/or human studies were included in our review if they addressed one or more of the following: (1) the effect of antidepressants on the microbiome, (2) the effect of the microbiome on antidepressants, and/or (3) the effect/correlation of antidepressant-induced microbiome changes on behavior. We excluded articles if any of the following criteria were met: (1) only in vitro models were used, (2) case reports, or (3) studies focused on mood or behavioral effects of drugs/compounds that are not classically defined as antidepressants (e.g., probiotics and other xenobiotics). Selection Process, Data Collection Process, and Data Items

Titles and abstracts were initially screened for potential inclusion independently by LCB and CAG according to the eligibility criteria. Full texts of the remaining articles were further screened by LCB and WVB according to the eligibility criteria. Disagreements on both the initial screening and full text screening were resolved by CAB. Only human/animal studies evaluating antidepressant-microbiome interactions were included. Case studies, in vitro studies, and non-antidepressant xenobiotics or probiotics were excluded. LCB extracted data from the included studies, including the model (for animal studies), sample size, sequencing techniques and primers, study (or experimental) design and duration, stool collection time, outcomes measures, sample characteristics, study drugs, experimental techniques/methods, assessment and follow-up procedures, main findings, interpretation of findings by study authors, and methodological limitations.

### 2.3. Risk of Bias Assessment

A risk of bias assessment for the included studies was conducted using the Risk of Bias in Non-randomized Studies of Interventions (ROBINS-I) tool [23]. The tool consists of three pre-intervention domains (bias due to confounding, bias in selection of participants into the study, and bias in classification of interventions) and four post-intervention domains (bias due to deviations from intended intervention, bias due to missing data, bias in measurement of outcomes, and bias in selection of the reported results). For each domain, studies were adjudicated as having a low, moderate, serious, or critical risk of bias according to established criteria [23]. The overall risk of bias for each study was equal to the most severe judgment across the seven domains assessed. 

### 2.4. Effect Measures and Synthesis Methods

Due to the inclusion of both animal and human studies and variability in study designs and reporting, main effects were summarized based on the individual data sources and were reported categorically into three thematic areas: (1) Antidepressant-dependent changes in gut microbiota composition, (2) Correlations between antidepressant-dependent gut microbiota changes on adverse events/behavior, and (3) Correlations between antidepressant-dependent gut microbiota changes and metabolites. Changes in gut microbiota included composition and diversity. Changes in adverse events/behavior were specific to correlations between these outcomes (side effects, response, remission in human studies or changes in specified behaviors in animal studies) and specific gut microbiota changes caused by antidepressants. Changes in microbial metabolites were correlated with changes in microbiota composition caused by antidepressants.

## 3. Results

**Figure 1:**
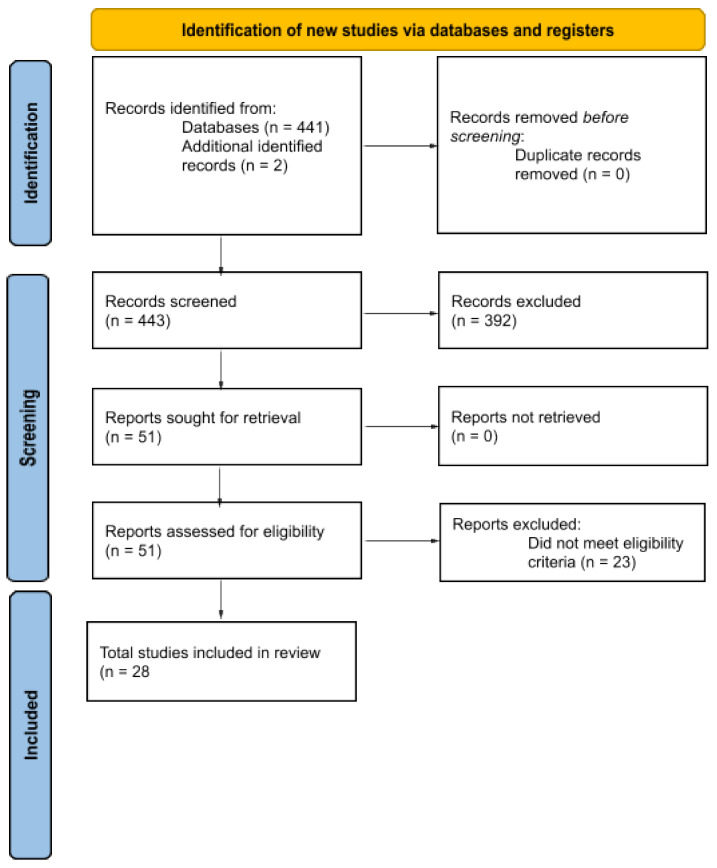
PRISMA flowchart [22] of included studies of antidepressant PMx in animals and humans.

The initial PubMed search resulted in 441 records and an additional review of review articles in the space and of included study reference sections yielded two additional records, totaling 443 records for Title and Abstract review (Figure 1). Of these, 392 were excluded for not meeting eligibility criteria, resulting in retrieval of 51 records for full text review. Twenty-three additional records were excluded for not meeting eligibility criteria, resulting in 28 studies that were included in the review (Table 1 and Table 2) [24]. The reviewed studies were heterogeneous with respect to experimental approach (human, animal), demographic characteristics (of participants in clinical studies), study medications, concomitant and previous medications, duration of medication exposure or treatment time, and sequencing tools. Most of the reviewed animal and human studies reported on antidepressant-associated changes in gut microbiota composition. Far fewer studies focused on how such changes correlated with adverse events, depressive symptoms, or depression-like behavior, or on correlations between antidepressant exposures and changes in microbial metabolites. The outcomes of preclinical and clinical studies are summarized in Supplementary Tables S1 and S2.

### 3.1. Animal Studies

Twenty studies evaluated PMx in animal models including mice (n = 15), rats (n = 5), and one in nematode worm *Caenorhabditis elegans* (n = 1), which will be summarized as animal studies hence forth. Experimental sample sizes ranged from three to 34 animals and study durations ranged from one day to 15 weeks. Most studies (n = 21) evaluated PMx in established animal models of depressive and anxiety-like behaviors to determine the effects of the antidepressants on the gut microbiota and/or behavior.

Klunemann found that bacteria in the microbiota of *C. elegans* can bioaccumulate duloxetine and mitigate the effect of the duloxetine on animal behavior [25]. Duloxetine and paroxetine reduced alpha diversity in mice in both Lukic and Dethloff studies, but buspirone rescued beta diversity in depression animal models [26,27,28]. 

Ketamine (n = 6) and fluoxetine (n = 6) were the most examined drugs in the included animal studies. The effect of fluoxetine on the microbiota varied across studies, but three studies reported lower levels of Bacteroidetes [29,30,31]. Zhang reported an increase in bacteroidetes. Lyte, Lukic, and Ramstein reported that fluoxetine perpetuated dysbiosis, but Sun and Zhang reported an increase in diversity with fluoxetine treatment [26,29,30,31,32]. When considering metabolite changes, Siopi found that treatment effects from fluoxetine are attenuated in mice transplanted with fecal microbiota from depressed mice by disrupting tryptophan metabolism [33]. Vuong found that fluoxetine treatment did not affect the microbiota of mothers, but did change transcription pathways in fetal brains, however, not through serotonergic pathways [34]. 

Interestingly, ketamine studies also reported different results related to the effect of ketamine on gut microbiota diversity. Both Getachew et al. and Qu et al. reported no change in the gut microbiota diversity and composition after treatment with ketamine, although, Qu et al. reported a greater effect of ketamine than lanicemine on the microbiota [35,36]. However, two studies demonstrated an increase in diversity with ketamine [37,38]. In regards to the enantimers of ketamine and its metabolite norketamine, Wang found that (S)-norketamine increased diversity more so than (R)-norketamine, but Yang reported that (R)-ketamine diversity changes were more similar to the control animals than (S)-ketamine [39,40].

The PMx of (es)citalopram was evaluated in four studies. While citalopram treatment in the Deng study did affect microbiota of depressed mice, it did not rescue dysbiosis compared to controls and Lukic showed that escitalopram reduced alpha diversity [26,41]. Duan showed differences in the microbiota of escitalopram responders compared to nonresponders where responders had an increase in bacteroidota (responses defined from behavioral tasks) [42]. Schmidtner also included escitalopram in studies, but as an adjunct to minocycline treatment where minocycline was shown to reduce microbial richness in rats [43]. Additionally, minocycline was found to reduce levels of Proteobacteria and levels of sarcosine and 2-aminoisobutanoic acid in the gut along with a decrease in inflammation [44].

Venlafaxine PMx was evaluated by both Lukic et al. and Serrano-Contreras et al. in which venlafaxine decreased alpha diversity while increasing beta diversity, but also decreased gut metabolites such as 3-(3-hydroxyphenyl)propionate, acetate, and propionate [26,45]. Desvenlafaxine, an active metabolite of venlafaxine, did not affect alpha diversity in the Lukic et al. study whereas fluoxetine, escitalopram, and duloxetine did [26]. For amitriptyline, Zhang et al. reported an increase in alpha diversity compared to healthy controls [32]. The PMx of buspirone was evaluated for changes in gut microbiota, inflammation, and behavior by Kim et al. [28]. The authors found that buspirone increased beta diversity, suppressed neuroinflammation, and improved anxiety/depressive-like behaviors in depressed mice. Notably, buspirone also perpetuated these changes in mice receiving fecal transplant from mice with depression-like behavior. 

### 3.2. Human Studies

Seven studies evaluated PMx in humans with a diagnosis of MDD and one study included individuals with MDD and comorbid irritable bowel syndrome (IBS) [46]. Individual studies ranged in sample size from 6 to 63 participants. Only three studies compared individuals with MDD to healthy controls (HC) [47,48,49]. All 7 of the human studies included in this review evaluated changes in microbiota composition after treatment with antidepressants. 

In a longitudinal study of 30 subjects with MDD and 30 healthy controls, flexibly-dosed escitalopram (up to 20 mg/day), α diversity was significantly higher at baseline between patients with depression and controls. A follow-up group consisted of patients with depression who achieved a symptomatic response to escitalopram, defined as a 50% or greater reduction (improvement) in depressive symptoms, as measured by the 17-item Hamilton Depression Rating Scale (HAM-D17). After treating with escitalopram, the α diversity in the follow-up group was not found to be significantly different than that of controls measured at baseline, suggesting that escitalopram was associated with favorable changes in the gut microbiota, with the caveat that microbial metabolites after escitalopram treatment in patients with depression still differed from that of controls [48]. 

Tomizawa and colleagues analyzed stool samples from 40 patients with MDD (n = 24), persistent depressive disorder (n = 8), or a co-occurring depressive and anxiety disorder (n = 12). Stool samples were collected at three separate time points while receiving naturalistic treatment with antidepressants from various classes, including SSRIs (n = 6), SNRIs (n = 6), mirtazapine (n = 3), tricyclics (n = 3), or antidepressant combinations (n = 16). Six patients with depression took no antidepressants. Antidepressants were not associated with changes in microbial diversity. However, nine antidepressant-treated patients also received concomitant antipsychotic medications, the doses of which were standardized using chlorpromazine equivalents. Exposure to antipsychotics was associated with reduced α diversity in a dose-dependent manner [50]. 

Dong et al. examined the composition and metabolic function of the gut microbiota in 63 patients who were hospitalized with a first episode of MDD and were treated with SSRIs or venlafaxine (mean 42.3 mg/day in fluoxetine equivalents) for 8 weeks. Thirty unaffected individuals served as healthy controls. At baseline, there were no significant differences in α diversity or β diversity between those with MDD and healthy controls across microbial community indices, although significant between-group differences in the relative abundances of specific microbial phyla, families, and genera were detected. There were no significant correlations between relative abundances of microbial species and the severity of depressive symptoms, as measured by the 24-item Hamilton Depression Rating (HAM-D24). Baseline fecal metabolites, however, differed between responders and non-responders in the MDD group, with the most significant differences observed for metabolites related to lipid metabolism. There were no significant differences in α diversity measured before and after antidepressant treatment in depressed subjects. Different patterns of change in the gut microbiome, particularly at the genus level, were reported for patients with MDD that had a positive response to antidepressants (defined as a 50%+ reduction in HAM-D24 total scores) and antidepressant non-responders [47]. 

Bharwani and colleagues also found differences in stool microbiota between responders and non-responders at various time points during treatment with citalopram and escitalopram in a cohort of 15 patients with MDD who received treatment over 6 months [51]. Greater phylogenetic diversity was observed in the responder group than in non-responders. At 3- and 6 months, 35 and 42 Operational Taxonomic Units (OTUs) were significantly different, respectively, between responders and non-responders. In the full sample, there were no significant changes in the gut microbiota during the course of treatment.

In a prospective 8-week study of 15 adults with depression and comorbid diarrhea-predominant irritable bowel syndrome (IBS, 6 received duloxetine treatment [up to 60 mg/day] and 9 received a commercially-available probiotic formula containing *Bifidobacterium longum, Lactobacillus acidophilus*, and *Enterococcus faecalis* strains), duloxetine treatment was associated with significant reductions in both self-reported depressive symptoms and IBS symptoms, and increased levels of *Faecalibacterium* [46]. Probiotic treatment was associated with a trend-level improvement in depressive symptoms and significant improvement in IBS symptoms. However, neither duloxetine nor probiotic treatment was associated with altered α diversity [46]. 

In a secondary analysis of data from a randomized trial of levomilnacipran for MDD in adults aged 60 years or older, Lee and colleagues found no significant differences in α-diversity or β-diversity between those who achieved symptomatic remission (HAM-D24 total score of 6 of less at 12 weeks post-baseline) and those who did not. However, greater abundance of *Faecalibacterium, Agathobacter* and *Roseburia* at baseline, relative to a reference frame, was associated with remission as a therapeutic outcome. Furthermore, significant changes in the gut microbiota at the genus level were observed with levomilnacipran treatment in remitters, but not in non-remitters [52].

Ye and colleagues analyzed fecal samples from 26 adults with MDD taken at baseline and after 4- and 8 weeks of treatment with vortioxetine (10 mg/day), and from 28 healthy control subjects at baseline. Fecal bacterial altered α diversity was higher in the MDD group than healthy controls. *Faecalibacterium* was negatively correlated with depression severity in individuals treated with vortioxetine, but there were no significant differences in gut microbiota diversity after treatment initiation [49]. 

### 3.3. Risk of Bias Assessment

A summary of the risk of bias assessment for each included study is provided in Table 3. None of the included studies were evaluated as having an overall low risk of bias. At the domain level, all studies were adjudicated as having a low risk of bias for classification of interventions and selection of the reported results. Moderate to serious risk of bias was detected for confounding (n = 27), selection of participants (n = 1), missing data (n = 2), and measurement of outcomes (n = 8). 

## 4. Discussion

To our knowledge, this is the first systematic review to investigate the PMx of antidepressants in the treatment of MDD in humans and animal models of human depression. Collectively, the current published findings suggest: (1) Animal and human studies have shown that antidepressants can alter the composition and metabolites of gut microbiota, (2) animal studies suggest that gut microbiota can alter the bioavailability of certain antidepressants, and (3) animal and human studies suggest that gut microbiota may modulate the clinical or modeled mood modifying effects of antidepressants [53].

This review, however, highlights the many unanswered questions related to the relationship between gut microbiota and clinical responses to pharmacotherapy, particularly for human MDD. For example, nearly all the reviewed human and animal studies assessed the effect of antidepressant exposures on the composition of gut microbiota. Indeed, several antidepressants have been shown to have antimicrobial activity, leading to direct effects on the composition of the gut microbiome and the potential for such changes to cause alterations in depression symptoms through the gut-brain axis [54]. However, there is a paucity of well-controlled studies that demonstrate a clear link between antidepressant-induced alterations in gut microbiota and antidepressant treatment responses in human patients. Additional studies are needed, therefore, to better define the potentially mediating or moderating effects of treatment-emergent changes in the composition of gut microbiota in the relationship between antidepressant exposure and improvement in depressive symptoms or medication side-effects.

Other research has focused on the effects of gut microbes on selected pharmacokinetic factors that may govern the availability of certain antidepressants. For instance, as shown in this review, the SNRI, duloxetine, can alter the composition of gut microbiota. However, by the opposite token, *C. elegans* has been recently shown to bioaccumulate duloxetine and, therefore, potentially mitigate the effect of the drug on behavior, suggesting a role of antidepressant bioaccumulation modulating drug exposure and therefore response to antidepressants [25]. Others have similarly shown that the metabolism of various drugs, including antidepressants, by microbes may also affect drug exposure, with the potential to influence therapeutic response and adverse events [55], similar to putative effects of drug-metabolizing cytochrome P450 isoenzymes in humans. In the case of antidepressants, a clear relationship between pharmacokinetic genes or serum drug concentrations and antidepressant response has been difficult to demonstrate with consistency [56,57,58]. Additional studies are needed to determine the degree to which microbial influences on drug availability impact therapeutic or adverse responses to antidepressants. 

Although antidepressant effects on broad “macro”-level changes in microbial biodiversity are of interest, homing in on antidepressant-associated changes in specific microbial species that may serve as pathobionts [microbes that can cause disease under certain conditions] in MDD may be particularly impactful as a focus of future work. Although it is highly unlikely that single bacterial species act alone to increase the risk of MDD, recent work has linked increased depression susceptibility with the heightened abundance of several bacterial species, including selected *Morganella, Myocobacterium, Bacteriodaceae, Bacteroides*, and *Segmented filamentous bacteria* species [59]. At present, it is unknown if successful treatment with antidepressants may have a relatively selective effect on the abundance of these particular species. In several of the reviewed studies, differential abundances of selected microbial species were observed between patients with MDD who responded well to antidepressants and those who did not or, in some cases, were predictive of eventual antidepressant response [47,51,52]. Although two of the reviewed studies seemed to converge on a possible role of *Faecalibacterium* for modulating the efficacy of some antidepressants [49,52], the specific findings across studies show quite a bit of heterogeneity, a likely reflection of the interindividual variability and temporal instability of the human gut microbiome [60].

An additional aspect of PMx that may be underappreciated is the effect of antidepressant withdrawal on the microbiome and, consequently, behavior. In the case of abusable substances, a recent review suggested that certain alterations in gut microbiota may be associated with reduced stress-associated symptoms including depression during withdrawal, thus reducing the risk of relapse [61]. To our knowledge, no studies have been conducted focusing on the relationship between changes or differences in the gut microbiome and the risk or intensity of rebound symptoms when certain antidepressants are suddenly stopped or doses are rapidly reduced. More importantly, in a recently-published randomized trial, over half (56%) of patients with recurrent MDD who stopped antidepressants relapsed during 52 weeks of follow-up, as compared to a relapse rate of 39% for those who continued their antidepressant [62]. We are unaware of any studies investigating the relationship between changes in the gut microbiome that may occur after antidepressants are stopped and the risk of depressive relapses or whether relapses despite continuing antidepressants may be similarly mediated by unfavorable changes in the gut microbiota.

The role of human genetics may also add yet another complexity to the gut-brain-PMx effect—an area that also awaits future study. For example, genetic variation in *DEFB1*, which codes for an antimicrobial peptide that plays a role in gut microbiome homeostasis, was recently associated with variation in plasma kynurenine concentration, a metabolite that was related to the severity of MDD symptoms in a cohort of depressed patients [63,64]. 

The field also lacks studies focused on gut microbiota-derived metabolites as biomarkers for antidepressant treatment effects. Such metabolites are directly produced by microbial species or are by-products of interactions between microbes and dietary substances or naturally produced host materials, and serve as key mediators for a variety of diseases [65]. Alterations in specific circulating or fecal metabolites may have direct effects on MDD disease risk or propensity for responding to particular antidepressants or may reflect favorable changes (from a depression risk or antidepressant response viewpoint) in the underlying gut flora [66].

### 4.1. Limitations

This systematic review was based on a primary search using PubMed. Although secondary identification of additional reports was performed by reviewing bibliographies of included studies and review papers, additional databases were not used. The individual studies reviewed herein are methodologically heterogeneous which would be expected to contribute to the conflicting results presented in this review. The exact time of microbiota samples were not reported in any of the included studies. Recent work suggests that gut microbiota, along with diet, undergo diurnal oscillations which affect gut metabolites and interactions with host cells [67]. The fact that even time of collection may affect the composition and function of gut microbiota composition needs to be considered as a variable in future studies. A majority of the studies reviewed here include animals rather than human subjects, limiting the applicability of results to human gut microbiota changes, effects, and behavior. Specifically, a majority of the animal models included herein were defined by anxiety-depressive behaviors in mice that were induced through stress models or stimulation of inflammation and may not truly reflect the symptomatology and pathology of depression in humans. Each of the studies varied by design and reported varying outcomes that ranged from the interaction of antidepressants and microbiota, effect of antidepressants on microbiota diversity, effect of antidepressants on microbiota metabolites, effect of antidepressants on microbiota function, effect of antidepressants on inflammation through antidepressant-gut microbiota interaction, and finally, the effect of antidepressant-gut microbiota interactions on adverse events or behavior making it difficult to conclude an overall effect of antidepressant PMx. All studies focused on reporting bacterial gut microbiota changes, and did not include other organisms such as fungi, parasites, archaea, and viruses. Notably, while most studies included universal primers for 16sRNA sequencing, the methods varied among studies leading to potential heterogeneity in reported bacterial results. An additional factor to consider when evaluating these studies is that most analyzed fecal/stool samples as a surrogate marker for gut microbiota composition and therefore may not actually reflect the true environment within the gastrointestinal system of the human or animal host. Finally, most human studies did not control for concomitant medications or comorbid conditions which may also affect outcomes. This review itself also includes limitations. We were limited in making conclusions around an overall effect of PMx of antidepressants due to the heterogeneity in study designs and outcomes reported. 

### 4.2. Future Studies

Future studies should focus on standardizing and controlling study variables and reporting so that the overall evidence can be evaluated to answer specific scientific and clinical questions. While these initial studies are promising, the field is far from utilizing PMx in personalized prescribing of antidepressant medications to improve outcomes. Additionally, incorporation of other markers including inflammation, imaging, genetics, and neurotransmitters may also increase the precision and understanding of the vast effect of PMx in an entire organism. 

Gut-microbiota composition may serve as a biomarker for medication selection, treatment outcome prediction, or the need for gut microbiota modulation in conjunction with medications. Baseline gut microbiota composition may be able to predict adverse events or efficacy to specific antidepressants therefore providing a potential clinical tool to personalize antidepressant selection to optimize treatment outcomes. Gut microbiota composition could also be analyzed over time with antidepressant treatment, as a surrogate to understand efficacy of the drug over time in conjunction with clinical measures. 

Although outside the scope of this review, an important related question is whether probiotic-associated changes in microbiota composition may modify the clinical effects of conventional antidepressants, ketamine, esketamine, or other antidepressant treatments in patients with MDD. From a clinical standpoint, probiotics are generally well tolerated and may be an attractive adjunct to antidepressants if antidepressant benefit can be consistently demonstrated and standardization of treatment can be achieved [68]. A recent systematic review evaluated the effect of probiotics, synbiotics, and prebiotics on depressive symptoms in 24 observational (n = 2817) and 19 interventional (n = 1119) studies and found that prebiotics had no effects and only synbiotics and probiotics had a modest effect, suggesting a role for microbiome modulation in treating depression but additional studies are needed [69]. Another review and meta-analysis of 7 randomized trials (n = 404 depressed subjects) of adjunctive or adjunctive probiotics for MDD (5 studies) or depressive symptoms (2 studies) showed a medium-to-large effect of probiotics added to antidepressants for reducing depressive symptoms (5 studies, n = 128 patients, SMD 0.83, 95% CI 0.49 to 1.17); however, similar effects on depressive symptoms were not observed for probiotic monotherapy (2 studies, n = 74 patients, SMD −0.02. 95% CI −0.34 to 0.30) [70]. These conclusions, however, were based on a small number of studies and varying formulations and, beyond the scope of the published review, dosing standards for probiotic supplementation for antidepressant-treated MDD are lacking. Furthermore, post-treatment change in the composition of gut microbiota was not included as an endpoint, limiting conclusions that can be made about gut microbial-based mechanisms as a mediator of treatment effects with adjunctive probiotics. Overall, the gut microbiota could be modulated through probiotics, synbiotics and prebiotics prior to, or in conjunction with, specific antidepressants to increase efficacy and decrease adverse events of the drug.

Interestingly, recent work in the probiotic space has focused on creating genetically modified bacteria in order to engineer production of specific metabolites or bacterial functional factors that can modulate the gut and therefore affect disease or the interaction of microbiota and drugs [71]. As the PMx space continues to grow, considerations will need to be taken concerning the role of “probiotics” and government drug regulation of live biotherapeutic products in efficacy and safety in humans [72]. 

## 5. Conclusions

The PMx of antidepressants field is expanding rapidly, however, study designs and outcomes reported vary greatly across publications. Clearly there lies an important interaction between the gut microbiota and antidepressants in potentiating adverse events and/or behavior, however, additional well controlled studies are needed to better characterize these interactions and understand how to implement PMx as a part of precision medicine in antidepressant treatment.

## Figures and Tables

**Table 1:** Animal studies of antidepressant PMx.

Author [Ref]	Pubmed ID	Model and Sample Size	Study Design	Study Duration	Stool Collection Time	Outcome Measure	Characteristics
Klunemann et al. [25]	34497420	*Caenorhabditis elegans* Each experiment = 50	Interventional observational, Controlled	1 h	N/A	Behavior	*C. elegans* N2 wild type *Escherichia coli* IAI1
Lukić et al. [26]	30967529	Mouse CTL = 9 FLU = 11 ESC = 12 VEN = 12 DUL = 11 DES = 12	Interventional observational, Controlled	3–4 wks	3 wks	Gut microbiota changes	Male BALB/c OlaHsd *Ruminococcus flavefaciens* 17 *Adlercreutzia equolifaciens* FJC-B9
Dethloff et al. [27]	32581888	Mouse PARO = 17 VEH = 17	Interventional observational, Controlled	1 and 2 wks	7 and 14 days (PARO = 17, VEH = 17)	Gut microbiota changes and gut metabolite changes	DBA/2J mice Depressed mouse model
Kim et al. [28]	33731795	Mouse CTL = 12 IS-treated = 12 EC-treated = 12	Interventional observational, Controlled	7 days	48 h after treatment	Gut microbiota changes and behavior	Male C57BL/6N mice IS or EC-induced model
Lyte et al. [29]	30643701	Mouse FLU = 10 CTL = 10	Interventional observational, Controlled	29 days	Baseline, days 15 and 29	Gut microbiota changes and behavior	Male CF-1 mice
Ramsteijn et al. [30]	31971855	Rat sMV-MC = 13 cMV-MC = 20 sMV-FLU = 25 cMV-FLU = 34	Interventional observational, Controlled	35 days	GD0 (before conception), GD7, GD14, PND2, PND7, PND14, and PND21	Gut microbiota and gut metabalomic changes	Serotonin transporter knockout (SERT−/−, Slc6a41Hubr) Wistar rats heterozygous SERT knockout (SERT+/−) female rats
Sun et al. [31]	31588192	Mouse CTL = 10 CUMS + VEH = 10 CUMS + FLU = 10	Interventional observational, Controlled	9 wks	7 wks	Gut microbiota changes and behavior	Male C57/6 mice
Zhang et al. [32]	33602895	Rat HC = 12 CUMS = 6 CUMS + AMI = 6 CUMS + FLU = 7	Interventional observational, Controlled	15 wks	9 (CUMS = 12 and HC = 12) and 15 (HC = 3, CUMS = 3, CUMS + AMI = 3, and CUMS + FLU = 3) wks	Gut microbiota changes, ARG changes, and behavior	Male pathogen-free Sprague–Dawley rats
Siopi et al. [33]	32187541	Mouse CTL = 10+ UCMS = 10+ CTL-tr = 10+ UCMS-tr = 10+ (all experiments had at least 10 animals in each group but varied)	Interventional observational, Controlled	8–9 wks	8–9 wks	Gut microbiota changes and behavior	Male C57BL/6J mice
Vuong et al. [34]	33979656	Mouse VEH = 3–6 FLU = 3–6 ABX + FLU = 3–6 ABX + VEH = 3–6	Interventional observational, Controlled	2 wks	E3.5, E6.5, E8.5, E11.5 and E14.5	Gut microbiota and brain gene transcription changes	Female SPF C57BL/6 J mice
Getachew et al. [35]	30579332	Rat KET = 5 SAL = 5	Interventional observational, Controlled	8 days	Day 8	Gut microbiota changes	Male Wistar rats
Qu et al. [36]	29147024	Mouse R-KET = 6 LAN = 6 SAL = 6 CTL = 6	Interventional observational, Controlled	15 days	Day 15 (3 days post-treatment)	Gut microbiota changes	Male C57BL/6 mice Male CD1 (ICR) mice
Huang et al. [37]	30528936	Mouse LPS + KET = 8 LPS + SAL = 8 CTL = 8	Interventional observational, Controlled	~25 h	~25 h after treatment	Gut microbiota changes and behavior	Male C57BL/6 mice LPS-induced inflammatory depression model
Wan et al. [38]	35594949	Mouse CTL = ~9 OVX + SAL = ~9 OVX + KET = ~9	Interventional observational, Controlled	6 wks	Day 43	Gut microbiota changes, bone marrow changes, and serum metabolite changes	Female C57BL/6 mice
Wang et al. [39]	34217782	Mouse CTL = ~9 SAL = ~9 RnKET = ~9 SnKET = ~9	Interventional observational, Controlled	28 h	28 h	Gut microbiota changes	Male C57BL/6 mice
Yang et al. [40]	29249803	Mouse CTL = 6 CSDS + SAL = 6 CSDS + SKET = 6 CSDS + RKET = 6	Interventional observational, Controlled	16 days	Day 16	Gut microbiota changes	Male C57BL/6 mice
Deng et al. [41]	33535879	Mouse CTL + PBS = 10 CTL + CIT = 10 CRS + PBS = 10 CRS + CIT = 10	Interventional observational, Controlled	5–6 wks	5–6 wks	Gut microbiota changes	Male C57BL/6 J mice CRS model
Duan et al. [42]	34016954	Mouse CTL = 8 CUMS + VEH = 8 ESC responder = 7 ESC nonresponder = 9	Interventional observational, Controlled	4 wks	Baseline and 4 wks of ESC	Gut microbiota changes, plasma metabolite changes, and behavior	Male C57BL/6 mice CUMS model
Schmidtner et al. [43]	31519869	Rat Sample sizes varied by experiment and was a minimum of 6 up to 15	Interventional observational, Controlled	22 days	Day 22	Gut microbiota changes and gut metabalomic changes	Male and female Wistar rats
Yang et al. [44]	32671421	Mouse CTL = 6–8 CUMS = 6–8 CUMS + MIN = 6–8 CUMS + IMI = 6–8	Interventional observational, Controlled	44 days	Day 44	Gut microbiota changes, adverse events, and gut metabolite changes	Male C57BL/6 mice
Serrano-Contreras et al. [45]	26895493	Rat VEN (22 mg/kg) + VEH = 18 VEN (112 mg/kg) + VEH = 18 VEH = 18	Interventional observational, Controlled	24 h	0 to 24 h after treatment	Gut metabolite changes	Female Wistar rats

**Table 2:** Human studies of antidepressant PMx.

Author [Ref.]	Pubmed ID	Model/Sample Size	Study Design	Study Duration	Stool Collection Times	Outcome Measure	Characteristics	Drug(s)
Zhang et al. [46]	30681503	Human DUL = 6	Interventional observational, uncontrolled	8 wks	Baseline and 8 wks	Gut microbiota changes, gut metabolite changes, plasma metabolite changes, and behavior	IBS and MDD 18–65 yo	DUL
Dong et al. [47]	35937875	Human MDD= 63 (20 males, 43 females) HC= 30 (10 males, 20 females)	Interventional observational case-controlled	8 wks	Stool collected at baseline and after 8 wks treatment	Gut microbiota changes and behavior	18–45 y/o First episode MDD Hospitalized BMI= 18.5–22.9	CIT, ESC, PARO, or VEN
Shen et al. [48]	34290352	Human MDD = 30 HC = 30	Interventional observational case-controlled	4–6 wks	Baseline and 4–6 wks after treatment	Gut microbiota changes and behavior	18–65 y/o Tx-naive, first episode MDD (no tx with antidepressant or antipsychotics) no recent effects on microbiome (ex: antibiotic usage)	ESC
Ye et al. [49]	34025474	Human HC = 28 MDD = 26	Interventional observational case-controlled	8 wks	Baseline, 4 wk, and 8 wks	Gut microbiota changes and behavior	18–50 yo	VOR
Tomizawa et al. [50]	32975292	Human depressive = 32 anxious = 8	Interventional observational, uncontrolled	3 wks	Baseline, 2wks, and 3 wks	Gut microbiota changes	Adult inpatient and outpatients with MDD and/or ANX 17 males and 23 females 33 stool samples at endpoint	AMI, AMO, SERT, PARO, ESC, DUL, MIL, VEN, MIR, APs, and anxiolytics
Bharwani et al. [51]	31958990	Human MDD = 15	Interventional observational, uncontrolled	6 months	Baseline, 3, and 6 month	Gut microbiota changes and behavior	18–60 y/o MDD dx Medication free at baseline	CIT and ESC
Lee et al. [52]	33757609	Human LVM = 4 (Remitters = 2) Placebo = 8 (Remitters = 3) Total Remitters = 5 Total Nonremitters = 7	Interventional observational controlled	12 wks	Baseline and 12 wks	Gut microbiota changes and behavior	Geriatric MDD dx Greater than 60 yo	LVM or Placebo

**Table 3:** Risk of Bias Assessment [23].

Study	Bias Due to Confounding	Bias in Selection of Participants into the Study	Bias in Classification of Interventions	Bias Due to Deviations from Intended Interventions	Bias Due to Missing Data	Bias in Measurement of Outcomes	Bias in Selection of the Reported Result	Overall Bias
Kluneman et al. [25]	Low	Low	Low	Low	Low	Moderate	Low	Moderate
Lukić et al. [26]	Moderate	Low	Low	Low	Low	Moderate	Low	Moderate
Dethloff et al. [27]	Moderate	Low	Low	Low	Moderate	Low	Low	Moderate
Kim et al. [28]	Moderate	Low	Low	Low	Low	Moderate	Low	Moderate
Lyte et al. [29]	Moderate	Low	Low	Low	Low	Moderate	Low	Moderate
Ramsteijn et al. [30]	Moderate	Low	Low	Low	Low	Low	Low	Moderate
Sun et al. [31]	Moderate	Low	Low	Low	Low	Low	Low	Moderate
Zhang et al. [32]	Moderate	Low	Low	Low	Low	Low	Low	Moderate
Siopi et al. [33]	Moderate	Low	Low	Low	Low	Low	Low	Moderate
Vuong et al. [34]	Moderate	Low	Low	Low	Low	Low	Low	Moderate
Getachew et al. [35]	Moderate	Low	Low	Low	Low	Low	Low	Moderate
Qu et al. [36]	Moderate	Low	Low	Low	Low	Low	Low	Moderate
Huang et al. [37]	Moderate	Low	Low	Low	Low	Moderate	Low	Moderate
Wan et al. [38]	Moderate	Low	Low	Low	Low	Moderate	Low	Moderate
Wang et al. [39]	Moderate	Low	Low	Low	Low	Low	Low	Moderate
Yang et al. [40]	Moderate	Low	Low	Low	Low	Low	Low	Moderate
Deng et al. [41]	Moderate	Low	Low	Low	Low	Low	Low	Moderate
Duan et al. [42]	Moderate	Low	Low	Low	Low	Low	Low	Moderate
Schmidtner et al. [43]	Moderate	Low	Low	Low	Low	Low	Low	Moderate
Yang et al. [44]	Moderate	Low	Low	Low	Low	Low	Low	Moderate
Serrano-Contreras et al. [45]	Moderate	Low	Low	Low	Low	Low	Low	Moderate
Zhang et al. [46]	Serious	Low	Low	Low	Low	Low	Low	Serious
Dong et al. [47]	Serious	Low	Low	Low	Low	Moderate	Low	Serious
Shen et al. [48]	Serious	Low	Low	Low	Low	Low	Low	Serious
Ye et al. [49]	Serious	Moderate	Low	Low	Low	Low	Low	Serious
Tomizawa et al. [50]	Serious	Low	Low	Low	Low	Serious	Low	Serious
Bharwani et al. [51]	Moderate	Low	Low	Low	Low	Low	Low	Moderate
Lee et al. [52]	Serious	Low	Low	Low	Moderate	Low	Low	Serious

## Data Availability

All collected data are in the publication and search results are available by contacting the corresponding author, L.C.B.

## Supplementary Materials

The following supporting information can be downloaded at: https://www.mdpi.com/article/10.3390/jpm13071086/s1, Table S1. Animal studies of antidepressant PMx; Table S2. Human studies of antidepressant PMx; Table S3. Abbreviations.
